# Hyperoxia increases the uptake of 5-fluorouracil in mammary tumors independently of changes in interstitial fluid pressure and tumor stroma

**DOI:** 10.1186/1471-2407-9-446

**Published:** 2009-12-17

**Authors:** Ingrid Moen, Karl J Tronstad, Odd Kolmannskog, Gerd S Salvesen, Rolf K Reed, Linda EB Stuhr

**Affiliations:** 1 Department of Biomedicine, University of Bergen, Bergen, Norway; 2 Heart and Circulatory Research Group, Haukeland University Hospital, Bergen, Norway

## Abstract

**Background:**

Hypoxia is associated with increased resistance to chemo- and radiation-therapy. Hyperoxic treatment (hyperbaric oxygen) has previously been shown to potentiate the effect of some forms of chemotherapy, and this has been ascribed to enhanced cytotoxicity or neovascularisation. The aim of this study was to elucidate whether hyperoxia also enhances any actual uptake of 5FU (5-fluorouracil) into the tumor tissue and if this can be explained by changes in the interstitium and extracellular matrix.

**Methods:**

One group of tumor bearing rats was exposed to repeated hyperbaric oxygen (HBO) treatment (2 bar, pO_2 _= 2 bar, 4 exposures à 90 min), whereas one group was exposed to one single identical HBO treatment. Animals housed under normal atmosphere (1 bar, pO_2 _= 0.2 bar) served as controls. Three doses of 5FU were tested for dose response. Uptake of [^3^H]-5FU in the tumor was assessed, with special reference to factors that might have contributed, such as interstitial fluid pressure (P_if_), collagen content, oxygen stress (measured as malondialdehyd levels), lymphatics and transcapillary transport in the tumors.

**Results:**

The uptake of the cytostatic agent increases immediately after a single HBO treatment (more than 50%), but not 24 hours after the last repeated HBO treatment. Thus, the uptake is most likely related to the transient increase in oxygenation in the tumor tissue. Factors like tumor P_if _and collagen content, which decreased significantly in the tumor interstitium after repeated HBO treatment, was without effect on the drug uptake.

**Conclusion:**

We showed that hyperoxia increases the uptake of [^3^H]-5FU in DMBA-induced mammary tumors *per se*, independently of changes in P_if_, oxygen stress, collagen fibril density, or transendothelial transport alone. The mechanism by which such an uptake occur is still not elucidated, but it is clearly stimulated by elevated pO_2_.

## Background

A tumor is comprised of cancer cells as well as stromal cells (fibroblasts, immune cells) that are embedded in an extracellular matrix (ECM) and nourished by vasculature. Because of irregular and tortuous tumor blood vessels with impaired blood flow and high proliferation rate, tumors have large hypoxic areas, especially in the central parts. It is now widely accepted that hypoxia induces tumor growth and enhances both radiation- and chemo-resistance of cancer cells [1].

Inefficiency of chemotherapy can partly be explained by development of multidrug resistance to different chemotherapeutic agents. However, the causes of hypoxia-mediated resistance are multifactorial. Some chemotherapeutic drugs require oxygen to generate free oxygen radicals that in turn induce cytotoxicity. Genetic and proteomic changes may have substantial effect, by altering proliferation kinetics, cell cycle position, inhibiting apoptosis and regulate angiogenesis and cellular metabolism [2]. However, drug resistance can also be caused by the inefficient transport of the anticancer drug into the tumor tissue. Solid tumors have a pathologically increased interstitial fluid pressure (P_if_) and a dense ECM that make transport of chemotherapeutic agents difficult [3-7]. Increased P_if _leads to decreased transcapillary transport, and thereby hinders efficient uptake of chemotherapy [8], while the fibrotic nature of the dense ECM in solid tumors have been shown to impede transport of molecules in the tumor interstitium, and thereby decrease the effect of cytostatic drugs [9-11]. Since P_if _can be lowered and the structure of the tumor interstitium can be altered, this could have the potential to enhance the efficiency of drug-based treatment of solid malignancies.

As hypoxia reduces the response of chemotherapeutic agents, we aimed to study the effect of enhanced oxygenation on drug-uptake in mammary tumors, by using hyperbaric oxygen (HBO). HBO increases oxygen tension and oxygen delivery to tissues independent of haemoglobin. This pO_2 _elevation has been shown to last for up to 60 min post HBO treatment [12]. Because of this, HBO has previously been used to enhance the pO_2 _in hypoxic tumor tissue, to potentiate the effect of radio- and photo-therapy in both clinical and preclinical trials and also the effect of some forms of chemotherapy like doxorubicin, alkylating agents and 5FU [13-16]. This effect has been ascribed as increased cytotoxicity of the tumor cells to the chemotherapeutic drug *in vitro *[16,17] and enhanced neovascularisation *in vivo *[18]. However, recent studies have shown reduced vascularisation after HBO treatment *in vivo *[19-21].

Therefore, the aim of the present study was to study if there is an effect of either a single (1) or repeated (4) treatments (each 90 min) of 2 bar pure oxygen on the uptake of radioactively labelled 5FU in the tumor tissue *per se*, and whether this is related to elevated pO_2_and micro-environmental factors like P_if_, collagen fibrils, transcapillary transport and oxygen stress. We would then be able to conclude that the HBO enhancement of chemotherapeutic effects, previously shown in the literature, is not only due to an enhanced sensitisation of the tumor cells.

## Methods

### Animals and tumor model

Female Sprague-Dawley rats were used. Mammary tumors (*adenocarcinomas*) were induced by *dimetyl-α-benzantracene *(DMBA) dissolved in olive oil and given to the rat by gavage at the age of 7 weeks at a dose of 16 mg (Møllegård, Denmark). The experiments were performed when the rats were 13-15 weeks old, having reached a bodyweight of approximately 250 g and developed one to three tumors along the mammary crest. Thus, n is equal to number of tumors, not the number of rats in these experiments. This was done to minimize the number of animals used. As tumors are very heterogenous, this was not considered a problem. We did not perform a power analysis prior to the experiments. The number of animals in each group is determined based on previous experience with rat experiments. However, this is always a general compromise between experimental accuracy and at the same time keeping the number of animals as low as possible. The previous variance in animal experiments has resulted in the minimum number of animals to n = 5. The animals were randomly allocated to the treatments groups, and none were excluded from the treatment procedure. Malondialdehyde and hydoxyproline experiments were performed blinded. As the main investigator performed both the HBO treatment and the rest of the follow-up experiments, blinding was difficult to accomplish. All the experiments were performed in accordance with recommendations of the Norwegian State Commission for Laboratory Animals and were approved by the local ethical committee.

### Hyperbaric hyperbaric chamber

A 30 L pressure chamber with an inner diameter of 25 cm, and an inner length of 65 cm was used. The chamber was supplied with pure O_2_, and the oxygen concentration (%) was monitored continuously by an oxygen meter (NUI, Bergen, Norway). After reaching 100% O_2 _within approximately 10 min, the pressure was raised over a period of approximately 3 min to 2 bar. The 2 bar pure oxygen atmosphere was maintained for a period of 90 min. To maintain >97% O_2 _atmosphere at all times, the chamber was flushed with pure oxygen for 3-5 min every 10-30 min depending on the number of animals in the chamber. The rats were then decompressed over a period of 10 min.

### Experimental groups and treatment design

The different experimental groups and their treatment details are given in Table 1. The repeated HBO treated rats were exposed to HBO on day 1, 4, 7 and 10, and the follow-up experiments were performed on day 11. The choice of this treatment protocol is due to a previous study [20], showing that 4 HBO treatments gave maximal growth-inhibitory effect. The single HBO treated group was only exposed to HBO on day 1 and measurements performed immediately after the exposure.

**Table 1:** The experimental groups.

Experimental groups	Gas	Ambient pressure	pO_2_	Number of exposures	Exposure time
**Control**	air	1 bar	0.2	-	-
**Repeated HBO treatment**	O_2_	2 bar	2.0	4	90 min
**Single HBO treatment**	O_2_	2 bar	2.0	1	90 min

### Dose response of 5FU

We tested 3 different doses of 5FU. Original dose (1.5 mg/kg), 1/3 (0.5 mg/kg) dose and 3× (4.5 mg/kg) dose 5FU was injected intraperitonally and tumor size was measured by a calliper on day 1, 4, 7 and 11, and estimated according to the formula: V = π/6 (a^2^·b), where 'a' denotes the shortest transversal diameter and 'b' the longest transversal diameter. Growth was calculated as growth change compared to day 1 (100%).

### Microdialysis

To determine the uptake of radioactively labelled 5FU ([^3^H]-5FU) (Nycomed Amersham, Buckinghamshire, UK) into the tumor tissue, microdialysis technique [22] was used, as modified in our laboratory [23]. After anaesthesia, the femoral vein was cannulated for injection of [^3^H]-5FU. One microdialysis probe (CMA/20, Microdialysis AB, Stockholm, Sweden) was placed in the jugular vein and one in the tumor. Both probes were connected to a pump (CMA-100, Microdialysis AB, Stockholm, Sweden) and the catheters were perfused with saline at a rate of 1 μl/min. The catheter and probes were left to stabilize and equilibrate for 60 min before sampling of dialysate. Sampling of dialysate from both tumor and plasma started immediately after injection of 0.2 ml 0.37 Mbq [^3^H]-5FU and fractions were collected every 10 min for a total of 70 min. The area under the curve (AUC) for the plasma and tumor was calculated as the product of counts per 10 min (cpm) for a total measurement period of 70 min. Transport of [^3^H]-5FU was expressed as AUC tumor divided by AUC plasma.

An additional, single HBO exposure group was performed to elucidate if the generally known enhanced effect of cytostatic agents in combination with HBO is due to the high pO_2 _during and immediately after HBO exposure. The rats were therefore operated before the single 90 min HBO exposure, and left to stabilize for 15 min post exposure. 0.2 ml 0.37 MBq [^3^H]-5FU was injected into the femoral catheter, and dialysate was sampled for the next 70 min, analogue to the standard microdialysis experiments.

### Measurement of interstitial fluid pressure (P_if_)

P_if _was measured using the wick-in-needle (WIN) technique [7,24]. Briefly, a standard 23-gauge needle, with a 2-4 mm long sidehole, filled with nylon floss and saline was inserted into the central part of the tumor and connected to a transducer dome through a PE-50 catheter. The fluid communication between the interstitium and the measuring system was confirmed by compression and decompression of the catheter (clamping). This caused a transient rise and fall in the pressure. A measurement was accepted when the pressure returned to pre-clamp value (± 1 mmHg).

### Determination of collagen content in the tumors

Hydroxyproline (to estimate collagen content) was determined in acid hydrolysates of the tumor tissue by a colometric method adapted from Woessner *et al*. [25].

### Oxygen stress measured by Malondialdehyd (MDA)

To evaluate oxidative stress on lipids and membranes (the status of oxidative damage) we measured the lipid peroxidation product MDA, in controls and HBO treated tumors. MDA was analysed by a spectrophotometric assay according to the manufacturer's protocol (Northwest Life Science Specialties, LCC, Vancouver). The level of thiobarbituric reactive substances (TBARS) was assessed in tissue homogenates (10% w/v prepared from frozen samples). As recommended for complex samples, the 3^rd^-derivative spectra (400-700 nm) were obtained, and MDA levels were calculated based on the peak value around 510-520 nm

### Volume calculations by the dilution principle

Extracellular volume (ECV) and plasma volume (PV) in tumor and skin was measured by radioactively labelled isotopes using the dilution principle as previously described [26]. Total tissue water (TTW) was calculated as: (wet weight-dry weight)/(dry weight). Samples were dried at 50°C until constant weight. The distribution volumes for ^51^Cr-EDTA (Institute of Energy Technology, Kjeller, Norway) and ^125^I-labeled human serum albumin (^125^I-HSA) (Institute of Energy Technology, Kjeller, Norway), measuring ECV and PV respectively, were calculated as plasma equivalent volumes, *i.e*. counts per min per mg of tissue divided by counts per min per ml of plasma. Both isotopes were given after functional nephrectomy by bilateral ligation of the renal pedicles via flank incision. ^51^Cr-EDTA (0.7 MBq) was injected into the jugular catheter and left to circulate for 85 min before injecting 0.05 MBq ^125^I-HSA. Blood samples were taken 5 min later, by heart puncture.

### Immunohistochemistry

The animals were sacrificed with saturated KCl during anaesthesia and the tumors were immediately dissected out and put into liquid N_2 _and then stored in -80°C.

Frozen tumor sections (20 μm) were used for studying lymph-vessel morphology. The sections were stained with an antibody against lymphatic vessel endothelial hyaluronan receptor-1 (LYVE-1) with the Avidin-Biotin Complex (ABC) method, using a commercially available kit (Vector Vectastatin Elite ABC Staining Kit, Vector Laboratories Inc). Diaminobenzidine (DAB) was used as a chromogen to visualize the lymph-vessels. Finally, the sections were counterstained with Richardsons dye. The sections were examined using a Nikon light microscope (THP Eclipse E600, Nikon Corporation, Tokyo, Japan) and the images were captured with a Nikon Digital Camera (DXM 1200F, Nikon Corporation, Tokyo, Japan).

### Statistics

All data were tested for normality prior to the choice of statistical analysis. Results were analysed statistically using two-way analysis with unpaired t-test comparing results from the groups. Paired t-test was used when comparing results within the same group of animals. Non-parametric Mann-Whitney test was used for the P_if _measurements, while ANOVA with Tukey post test was used to compare the results from the microdialysis. A value of p < 0.05 was considered statistically significant.

## Results

### Dose response of 5FU

**Figure 1:**
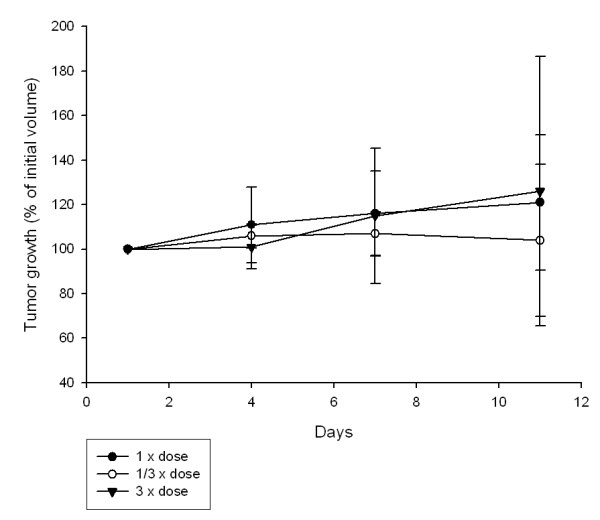
**The effect of three different doses of 5-fluorouracil (5FU) on tumor growth, original dose (1.5 mg/kg), 1/3 (0.5 mg/kg) dose and 3× (4.5 mg/kg) dose**. Tumor growth is measured as % of initial volume. Mean ± SD.

To examine if different doses of the chemotherapeutic drug might influence the effect on tumor growth, dose response of 3 different doses were tested. However, there was no significant difference in tumor growth inhibition between the three different doses of 5FU (Figure 1). (n = 5 for each group).

### Uptake of [^3^H]-5FU

Microdialysis was used to examine if oxygenation of the tumor after HBO treatment influenced the direct uptake of the chemotherapeutic drug into the tumor tissue. Microdialysis showed no increase in uptake of [^3^H]-5FU into the tumor tissue 24 hours after the last repeated HBO treatment (n = 8) compared to control (n = 7). However, immediately after the single HBO treatment (n = 5) there was a significant increase in uptake (p < 0.05) (Table 2).

**Table 2:** Uptake of [^3^H]-5-fluorouracil (5FU), interstitial fluid pressure (P_if_), collagen content and malondialdehyd (MDA) levels in controls and after repeated or single hyperbaric oxygen exposure (HBO).

	Control	Repeated HBO	Single HBO
**Uptake of [^3^H]-5FU** (AUC tumor/AUC plasma)	0.17 ± 0.12 (n = 7)	0.20 ± 0.11 (n = 8)	0.42 ± 0.21 * (n = 5)
**P_if_** (mmHg)	5.5 ± 2.6 (n = 9)	2.3 ± 2.7* (n = 11)	2.5 ± 2.9* (n = 17)
**%collagen of dry weight**	11.6 ± 5.0 (n = 6)	5.0 ± 1.1* (n = 4)	
**MDA levels** **(**nmol/mg prot)	0.09 ± 0.01 (n = 5)	0.15 ± 0.11 (n = 5)	0.09 ± 0.02 (n = 5)

Mean ± SD.

* p < 0.05 vs control

### Hyperoxia lowers interstitial fluid pressure (P_if_)

As elevated P_if _has been proposed to inhibit the transport and effect of chemotherapy, we wanted to elucidate if this was the case also for DMBA induced mammary tumors. The P_if _is usually high in solid tumors, reaching 5.5 ± 0.9 mmHg in the control tumors (n = 9) (Table 2). However, the average P_if _after both single (n = 17) and repeated HBO (n = 13) treatment was significantly reduced (p < 0.05) compared to control.

### Hyperoxia influences collagen fibrils

A dense collagen network has been proposed to hinder efficient transport of chemotherapeutic drugs. Therefore we have elucidated collagen content in both control and HBO treated tumors. The amount of hydroxyproline was significantly lower in the HBO treated tumors compared to controls, indicating a corresponding decrease in collagen content (Table 2).

### Hyperoxia and oxygen radicals

As hyperoxia might induce elevated oxygen radical levels, it was desirable to measure if this could have influenced the effect of chemotherapy after HBO treatment. Measurements of MDA-levels showed no significant differences in oxygen radicals compared to control (n = 5), neither in the acute (n = 5) nor the repeated (n = 5) HBO treated group (Table 2). The higher mean value in the repeated group is due to one diverging value. Discarding this value would give an identical mean as to the two other groups.

### Extracellular fluid volume, plasma volume and total tissue water

Since P_if _in the HBO treated tumors was lowered, we assumed that this could influence the fluid distribution. However, there was no statistically significant difference in ECV, PV or TTW in either the tumor or skin tissue between control (n = 9) and repeated HBO treated rats (n = 8) (Table 3).

**Table 3:** The extracellular volume (ECV), plasma volume (PV) and total tissue water (TTW) in skin and tumor in control and after repeated hyperbaric oxygen (HBO) exposure.

	ECV (ml/g dry weight)		PV (ml/g dry weight)		TTW (ml/g dry weight)	
	
	Skin	Tumor	Skin	Tumor	Skin	Tumor
**Control** (n = 9)	0.55 ± 0.12	1.00 ± 0.27	0.007 ± 0.004	0.07 ± 0.04	1.44 ± 0.09	3.73 ± 0.45
**HBO** (n = 8)	0.50 ± 0.18	0.85 ± 0.47	0.004 ± 0.003	0.06 ± 0.03	1.45 ± 0.2	4.05 ± 0.9

Means ± SD.

### Hyperoxia influenced tumor lymphatics

**Figure 2:**
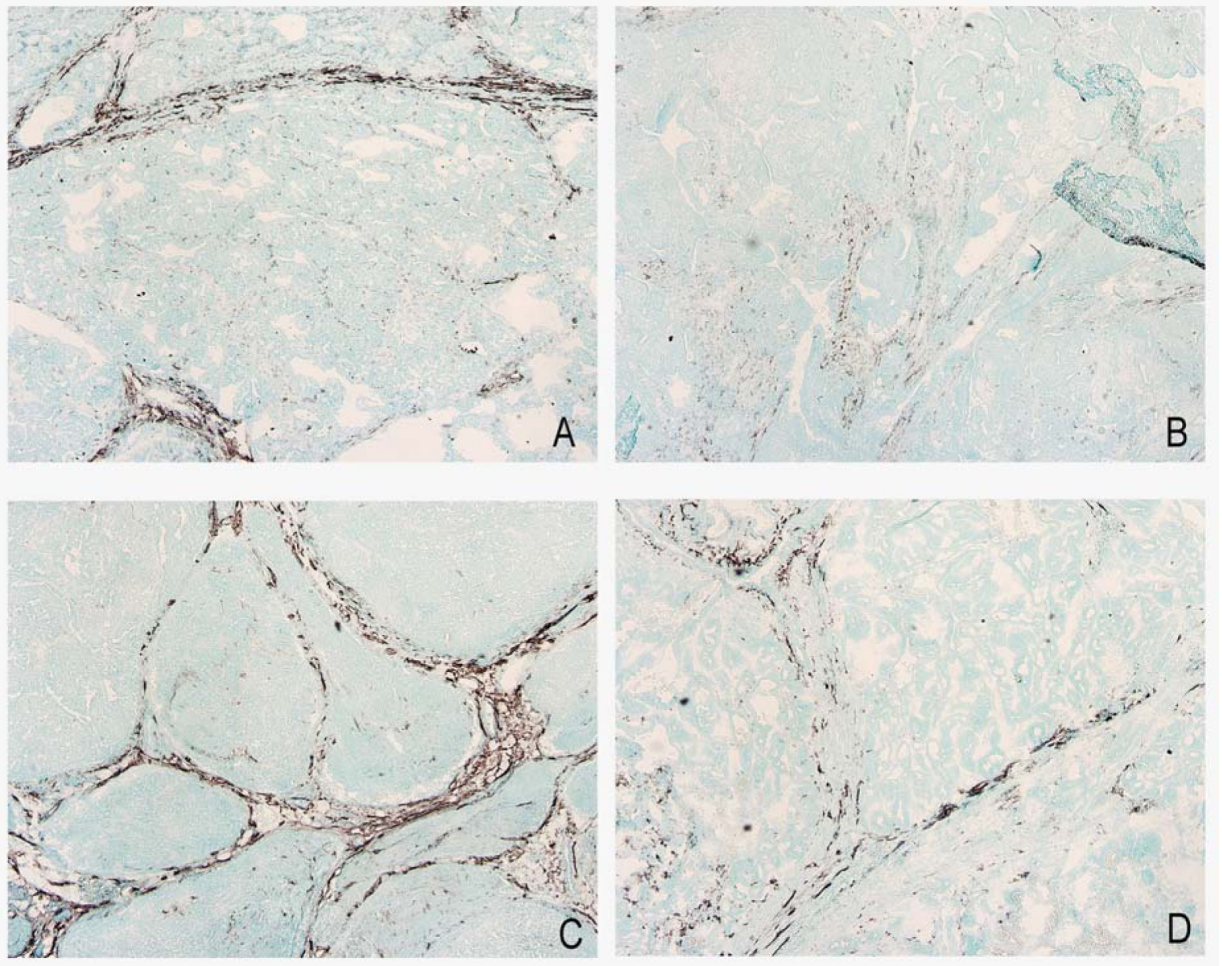
**Lymph vessels stained with LYVE-1 in two different control tumors (A and C) and hyperbaric oxygen (HBO) treated tumors (B and D)**. The staining was performed concomitant in the two groups. Note disintegration of lymphatics after HBO treatment, and seemingly anatomically normal lymphatics in untreated control tumors.

As the amount and functionality of lymph vessels influence both P_if_, fluid distribution and possibly the transport of chemotherapy, we stained lymph vessels to elucidate possible changes after HBO. Staining with LYVE-1 showed continuous lymphatic vessels in the mammary control tumors, located in the connective tissue close to larger blood vessels. After HBO treatment, however, the lymphatic vessels are disintegrated and no longer continuous (Figure 2).

## Discussion

Stuhr *et al*. [14] demonstrated increased effect of the commonly used cytostatic drug 5FU in DMBA-induced mammary tumors, when injected intraperitoneally immediately before each HBO treatment. Dose-response experiments in this study showed no statistical difference between 1/3×, 1× and 3× dose of 5FU (Figure 1), indicating that it must be the HBO treatment that potentiated the effect of the chemotherapeutic drug. We therefore wanted to investigate if there is an effect of either a single or repeated treatment of 2 bar pure oxygen on the uptake of radioactively labelled 5FU in the tumor tissue *per se*. We have concluded that the potentiation of the chemotherapeutic effect after HBO is not only due to an enhanced cytotoxicity of the tumor cells, as has been previously postulated in the literature [15,27,28], but also due to an direct increase in uptake of the chemotherapeutic drug.

Successful delivery of systemically administered cytostatic agents to the tumor tissue requires that the drug must reach the target cells in optimal dosage and in addition be effective in the tumor microenvironment. After a molecule has been moved out of the blood vessels and into the interstitium, further transport is heavily influenced by the organization and conductivity, and thereby the composition of the interstitium. Several investigators have shown increased uptake of cytostatic agents after decreased tumor P_if _[8,29-32]. In a recent review, Heldin *et al*. [8] suggest that increased P_if _contributes to a decreased capillary transport in tumors, to hinder efficient uptake of drugs into the tumor tissue. Additionally, the composition and organisation of the ECM, cell-cell interactions, and the tumor structure also affect drug penetration [33]. Eikenes *et al*. [11] proposed that the structure of the ECM is the single most important factor for macromolecule uptake. In accordance with this, Netti *et al*. [9], found an unexpected correspondence between transport resistance and the mechanical stiffness of the ECM, with an extended collagen network in the more penetration-resistant tumors. Also, others have shown that tumors with high collagen density and small interstitial space have lower drug penetration [34,35]. Furthermore, increased blood flow and blood vessel permeability are factors that are important in drug-uptake [36]. Thus, we chose especially to investigate the relation between uptake of the cytostatic drug, transendothelial transport, tumor P_if _and the collagen fibril network.

We found a significant reduction in tumor P_if _after both acute and repeated HBO treatment, and expected that this would be reflected in an increased uptake of [^3^H]-5FU after both treatments. This is, however, not the fact in our study, where the uptake of [^3^H]-5FU was significantly increased after single HBO treatment, but not after the repeated HBO treatment. The cpm-ratio between tumor and plasma was stable over the 70 min sampling-period during microdialysis for the single HBO treatment, indicating a constant condition determining the uptake. The increased uptake of the drug into the tumor tissue, after single HBO exposure is most likely influenced by increased pO_2_. Previous studies reported enhanced pO_2 _for up to one hour after HBO treatment [12,37]. Since the repeated HBO treated animals were measured 24 hours after the last HBO treatment, the pO_2 _is normalized and any effect of pO_2 _will not be measurable. In the single HBO experiments, however, the pO_2 _is still high. The repeated HBO treatment probably induces more long-term changes in the tumor tissue that influences P_if_. The reduction in collagen may be involved in the reduction of P_if _over time. Stromal fibroblasts are able to exert tension on the collagen microfibrillar network through the collagen binding integrins [38]. When the amount of collagen fibrils is reduced, as shown with hydroxyproline quantification, this effect would not be as pronounced, and the P_if _might be reduced. Thus, it seems like repeated HBO treatment have reduced tumor P_if _by reducing collagen content and density, most likely by enhancing breakdown of collagen.

Tumor vessels are hyperpermeable due to up-regulation of vascular mediators such as nitric oxide, bradykinin as well as anatomic defects like large gap junctions between adjacent cells and lack of pericytes [36,39]. An explanation for the lack of uptake of [^3^H]-5FU in the repeated HBO treated tumors, might be the proposed mechanism by Lee *et al*. [40] of "normalization" of the abnormal structure and function of the tumor vasculature after anti-angiogenic treatment. We have previously shown that repeated HBO induces an anti-angiogenic effect in the DMBA induced tumors [19,21]. Lee *et al*. [40] suggested that it is the quality of the vascular organization and not just the quantity of the vessels that determines the vessel function, and that the loss of endothelial cells would reduce the tortuousity of vessels or eliminate them altogether. Later, several preclinical and clinical studies [41,42] have shown that the "normalized" vasculature after anti-angiogenic therapy had less leaky and tortuous vessels, with more normal basement membranes and better pericyte coverage, and that these structural changes were accompanied by "normalization" of the tumor microenvironment. This modification in vascular architecture would decrease vascular permeability and reduce flow resistance and hence lower both mean venous pressure and P_if _[8]. Experimental studies have demonstrated that anti-angiogenic therapy can decrease the overall distribution of large macromolecules such as antibodies for instance [43,44] and decrease blood perfusion [45]. Thus, since we have previously shown that HBO has an anti-angiogenic effect on DMBA induced tumors after repeated HBO treatment [19], this will together with a possible reduced capillary permeability, impede transendothelial transport of [^3^H]-5FU, even though P_if _is lowered.

In normal tissue, the interstitial fluid volume is kept fairly constant by several mechanisms [46], such as lymph flow and adjustment of pressures acting across the capillary wall [47]. LYVE-1 surprisingly shows continuous lymph vessels in our control tumors, but dissolved lymph vessels after repeated HBO treatment. However, Fukumura *et al*. [48] stated that even though the structures with lymphatic endothelial marker are present in tumors, they probably do not transport fluid or macromolecules. This, together with the observed lowering of tumor P_if _after repeated HBO treatment, should lead to an increase in ECV. Nevertheless, ECV, PV and TTW did not change in tumors after repeated HBO treatment compared to control, indicating that neither the lowered P_if _nor the disintegrated lymphatics after HBO contribute to the unchanged ECV found in the present study. As already mentioned, the possible "normalization" of the tumor vasculature, after repeated HBO treatment, is expected to normalize capillary permeability, and thereby prevent any increase in ECV, even though P_if _is decreased.

Reactive oxygen species and oxidative stress are considered to be important in several aspects of malignancies, both in tumor development as well as therapeutic strategies such as radiotherapy [49]. Since changes in oxygen concentration may affect the production of its reactive derivatives in the tumor, it is relevant to suggest that antitumor effects of HBO may be due to oxidative stress. Furthermore, as stated in the introduction, some chemotherapeutic drugs require oxygen to generate free radicals that induce cytotoxicity. However, our measurements of lipid peroixidation (MDA) did not indicate any significant effect on the oxidative status of the tumor tissue. Oxidative stress is therefore not likely to be involved in the potentiated effect of 5FU after HBO.

Most solid tumors depend on increased rates of glycolysis to satisfy their energy demand [50]. This leads to decreased pH in the tumor microenvironment due to excessive lactate secretion. Hyperoxic treatment enhances oxygenation of the tumor tissue, and may therefore promote a reversion from the anaerobic metabolism back to non-tumorigentic, oxidative metabolism and thereby increased pH [21]. A less acidic microenvironment may therefore have a positive effect on the cytotoxicity of chemotherapy. However, although increased cytotoxicity has previously been postulated to be the main determinant of the enhanced effect of chemotherapy after HBO treatment in addition to enhanced neovascularisation, the present study has shown that it is also due to an active uptake of the drug into the tumor tissue.

In **conclusion**, we showed that hyperoxia increases the uptake of [^3^H]-5FU in DMBA-induced mammary tumors, independently of changes in P_if_, collagen fibril density, or transendothelial transport alone, as one could expect from the literature. The mechanism by which such an uptake occurs is clearly stimulated by elevated pO_2_, but still not elucidated.

## Competing interests

The authors declare that they have no competing interests.

## Authors' contributions

IM carried out the HBO treatments, the microdialysis, the P_if_measurements, the volume experiments and the LYVE staining, in addition to drafting the manuscript. KJT performed the MDA experiments. OK carried out the hydroxyproline measurements. GS performed the dose response experiments and assisted on the microdialysis and volume experiments. RKR and LS participated in the study design, interpretation of data and manuscript drafting. All authors read and approved the final manuscript.

## Pre-publication history

The pre-publication history for this paper can be accessed here:

http://www.biomedcentral.com/1471-2407/9/446/prepub
